# Association of *RTEL1* gene polymorphisms with stroke risk in a Chinese Han population

**DOI:** 10.18632/oncotarget.22980

**Published:** 2017-12-05

**Authors:** Yi Cai, Chaosheng Zeng, Qingjie Su, Jingxia Zhou, Pengxiang Li, Mingming Dai, Desheng Wang, Faqing Long

**Affiliations:** ^1^ Department of Neurosurgery, The Second Affiliated Hospital of Hainan Medical College, Hainan 570311, China

**Keywords:** single nucleotide polymorphisms (SNPs), RTEL1, telomere, stroke, case-control study

## Abstract

We investigated the associations between single nucleotide polymorphisms (SNPs) in the regulator of telomere elongation helicase 1 (*RTEL1*) gene and stroke in the Chinese population. A total of 400 stroke patients and 395 healthy participants were included in this study. Five SNPs in *RTEL1* were genotyped and the association with stroke risk was analyzed. Odds ratios (ORs) and 95% confidence intervals (95% CIs) were calculated using unconditional logistic regression analysis. Multivariate logistic regression analysis was used to identify SNPs that correlated with stroke. Rs2297441 was associated with an increased risk of stroke in an allele model (odds ratio [OR] = 1.24, 95% confidence interval [95% CI] = 1.01–1.52, *p* = 0.043). Rs6089953 was associated with an increased risk of stroke under the genotype model ([OR] = 1.862, [CI] = 1.123–3.085, *p* = 0.016). Rs2297441 was associated with an increased risk of stroke in an additive model (OR = 1.234, 95% CI = 1.005, *p* = 0.045, Rs6089953, Rs6010620 and Rs6010621 were associated with an increased risk of stroke in the recessive model (Rs6089953:OR = 1.825, 95% CI = 1.121–2.969, *p* =0.01546; Rs6010620: OR = 1.64, 95% CI = 1.008–2.669, *p* =0.04656;Rs6010621:OR = 1.661, 95% CI = 1.014–2.722, *p* =0.04389). Our findings reveal a possible association between SNPs in the *RTEL1* gene and stroke risk in Chinese population.

## INTRODUCTION

Stroke is a major cause of death and disability worldwide as well as a major burden on public health worldwide [1]. Within the globe in 2010, roughly 10% of the 52769700 deaths and about 4% of the 2490385000 disability adjusted life years (DALYs) were due to stroke, with developing countries becoming more severe than in developed countries [2]. Globally, stroke is projected to be the fourth most common cause of premature death and disability by the year 2020 [2]. Stroke is a multifactorial disease associated with a variety of factors. In previous studies, hypertension, diabetes, obesity, smoking and advanced age were identified as risk factors for stroke [3]. Through the study of twins and family history, family inheritance is also closely related with stroke [4]. Simultaneously, Genetics as a risk factor for stroke have also been confirmed, and some genetic polymorphisms such as MTHFR, ApoE [5], P-selectin and interleukin-4 gene [6] have been shown to be associated with the risk of stroke.

Telomere, as a protective structure at the end of chromosome, plays a crucial role in maintaining the stability of the genome. The shortening of telomere length is related to cell senescence and apoptosis. Many studies have confirmed that telomere length is associated with stroke risk, shortening telomere length increases the risk of stroke [7–9]. Genetic polymorphisms of telomere-related genes such as ACYP2, TSPYL6, and TERT have been reported to be associated with stroke [10, 11]. Regulator of telomere elongation helicase 1(*RTEL1*) belongs to the superfamily 2 (SF2) helicase [12], is an essential helicase that plays an important role in maintaining telomere length and genomic stability [12, 13] *RTEL1* gene, located in chromosome 20q13.3, plays an important role in DNA repair and apoptosis [14]. The single nucleotide polymorphism (SNP) of *RTEL1* gene has been confirmed to be associated with telomere-related diseases such as Hoyeraal Hreidarsson Syndrome [15], glioma [16], lung cancer [17] and atopic dermatitis [18]. However, so far, no studies have investigated the association between SNPs in the *RTEL1* gene and the risk of stroke. In this case-control study, we genotyped five SNPs in *RTEL1* gene: rs6089953, rs6010620, rs6010621, rs4809324 and rs2297441, and performed a comprehensive association analysis to identify SNPs associated with the risk of stroke in Chinese population.

## RESULTS

### Participant characteristics

A total of 400 patients with stroke and 395 healthy individuals were enrolled in the study. The demographic characteristics of the participants are shown in Table 1.The Pearson’s test showed that there was no statistical difference in sex distribution between case group and control group (*p* > 0.05). The mean age of the participants was 66.83 years in the case group and 48.67 years in the control group.

**Table 1:** Demographic characteristics of the patients with stroke and control individuals

Characteristic		Cases (*N* = 400)	Controls (*N* = 395)	*P*-value
gender	female	137 (34.3%)	152 (38.5%)	0.215
	male	263 (65.8%)	243 (61.5%)	
Mean age ± SD		66.83 ± 11.637	48.67 ± 11.059	< 0.001

SD: Standard deviation.

*P* value of gender was calculated by Welch’s *t* test

*P* value of age was calculated by Pearson’s χ^2^ test.

### Association between SNPs in the *RTEL1* gene and Stroke

The detailed data of the SNP of the *RTEL1* gene and the associations between various SNPs and stroke risk are shown in Table 2. We performed the Hardy Weinberg equilibrium test on the 5 SNPs investigated, and found that all 5 SNPs were in Hardy Weinberg equilibrium in the control subjects (*p* > 0.05). Rs2297441 was associated with an increased risk of stroke in an allele model(odds ratio [OR] = 1.24, 95% confidence interval [95% CI] = 1.01–1.52, *p* = 0.043).While in the other four SNPs(Rs6089953, Rs6010620, Rs6010621, Rs4809324), no associations were observed between the alleles and stroke risk.

**Table 2:** Basic information of candidate SNPs in this study

SNP-ID	Band	Position	Role	Alleles A/B	HWE	MAF		OR	95% CI		*P* value
						Case	Control				
rs6089953	20q13.33	62291008	Intron	G/A	0.457	0.326	0.282	1.23	1.00	1.53	0.054
rs6010620	20q13.33	62309839	Intron	G/A	0.900	0.308	0.276	1.17	0.94	1.45	0.166
rs6010621	20q13.33	62310872	Intron	G/T	0.801	0.301	0.272	1.15	0.93	1.43	0.200
rs4809324	20q13.33	62318220	Intron	C/T	1.000	0.150	0.141	1.08	0.82	1.43	0.577
rs2297441	20q13.33	62327582	Upstream	A/G	1.000	0.383	0.334	1.24	1.01	1.52	0.043

SNPs: Single nucleotide polymorphisms; MAF: Minor allele frequency; HWE: Hardy-Weinberg equilibrium; OR: Odds ratio; CI: Confidence interval. A :Minor alleles. B: Major alleles.

**Figure 1:**
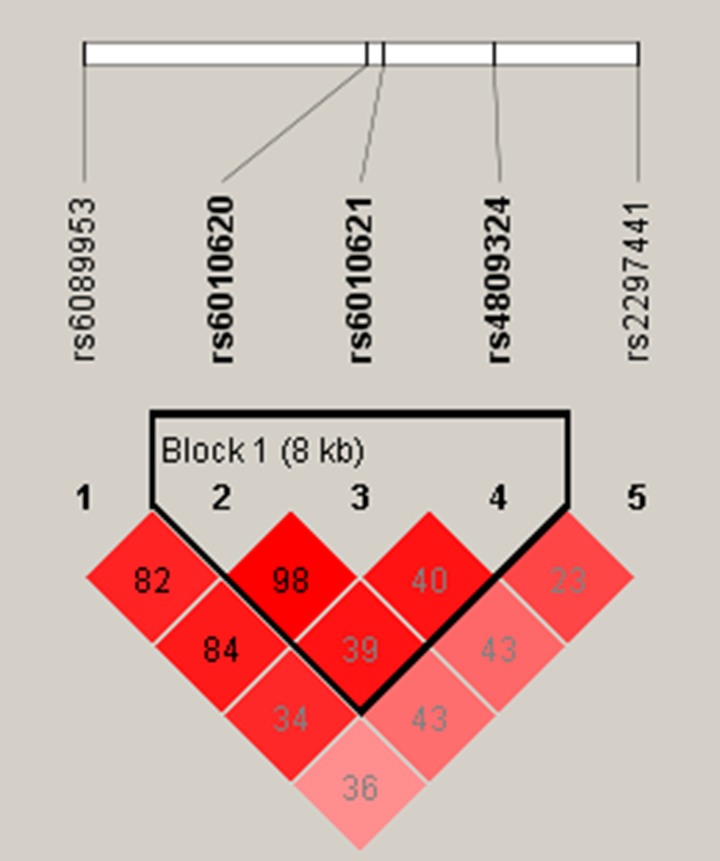
Haplotype block map for all the SNPs of the *RTEL1* gene

We further assessed the association between each SNP and stroke risk using the four models in the unconditional logistic regression analysis: genotype, additive, dominant, and recessive models (Tables 3 and 4). Rs6089953 was associated with an increased risk of stroke under the genotype model (odds ratio [OR] = 1.862, 95% confidence interval [95% CI] = 1.123–3.085, *p* = 0.016) (Table 3). Rs2297441 was associated with an increased risk of stroke in an additive model (OR = 1.234, 95% CI = 1.005, *p* = 0.045, Rs6089953, Rs6010620 and Rs6010621 were associated with an increased risk of stroke in the recessive model (Rs6089953:OR = 1.825, 95% CI = 1.121–2.969, *p* = 0.01546; Rs6010620:OR = 1.64, 95% CI = 1.008–2.669, *p* = 0.04656;Rs6010621:OR = 1.661, 95% CI = 1.014–2.722, *p* = 0.04389). We then evaluated the relationship between SNP haplotypes and stroke, no positive results were observed (Table 5, Figure 1). All of the above results were obtained by the Wald test.

**Table 3:** Single loci association with stroke

SNP-ID	Dominant				Recessive				Additive			
	OR	95% CI		*P*	OR	95% CI		*P*	OR	95% CI		*P*
rs6089953	1.16	0.88	1.54	0.289	1.83	1.12	2.97	0.015	1.23	0.99	1.52	0.057
rs6010620	1.09	0.83	1.44	0.544	1.64	1.01	2.67	0.047	1.16	0.94	1.43	0.176
rs6010621	1.07	0.81	1.41	0.640	1.66	1.01	2.72	0.044	1.15	0.93	1.42	0.210
rs4809324	1.03	0.75	1.41	0.876	1.87	0.74	4.73	0.188	1.08	0.82	1.42	0.583
rs2297441	1.27	0.96	1.68	0.100	1.42	0.94	2.16	0.099	1.23	1.01	1.52	0.045

SNPs: Single nucleotide polymorphisms; OR: Odds ratio. CI: Confidence interval. *P* value was calculated by Wald test. ^*^p < 0.05 indicates statistical significant.

**Table 4:** The association between the single-nucleotide polymorphisms and stroke in Genotype model

SNP-ID	Genotype	Case (*N*)	Control (*N*)	OR	95% CI		*P*
rs6089953	GG	49	28	1.86	1.12	3.09	0.016
	GA	163	166	1.05	0.78	1.40	0.771
	AA	188	200	-	-	-	0.051
rs6010620	GG	46	29	1.63	0.99	2.70	0.056
	GA	154	160	0.99	0.74	1.33	0.954
	AA	200	206	-	-	-	0.138
rs6010621	GG	45	28	1.64	0.98	2.73	0.058
	GT	151	159	0.97	0.72	1.30	0.830
	TT	204	208	-	-	-	0.128
rs4809324	CC	13	7	1.85	0.73	4.71	0.196
	CT	94	97	0.97	0.70	1.34	0.835
	TT	292	291	-	-	-	0.412
rs2297441	AA	60	44	1.56	1.00	2.44	0.050
	AG	184	176	1.20	0.89	1.61	0.242
	GG	153	175	-	-	-	0.130

OR: odd ratio; CI: confidence interval; *p* value was calculated by Wald test. ^*^p < 0.05 indicates statistical significance.

**Table 5:** Haplotype frequency and their association with stroke in case and control subjects

SNPs	Haplotype	Frequency%		OR	95% CI		Wald	*P*
		case	control					
rs6010620|rs6010621|rs4809324	GGC	0.1454	0.1367	1.072	0.8113	1.415	0.4872	0.6261
	GGT	0.1554	0.1354	1.172	0.8875	1.548	1.119	0.2631
	ATT	0.688	0.7203	0.8612	0.6966	1.065	−1.381	0.1672

SNPs: SNPs forming the haplotype

Wald: Z statistics of wald test for measuring odds ratio departure from 1

P: *P* value calculated by Wald test. *P*-value < 0.05 indicates statistical significance.

## DISCUSSION

In this study, we investigated the associations between SNPs in the *RTEL1* gene and stroke risk in the Chinese population. Among the five SNPs we selected, four SNPs (rs6089953, rs6010620, rs6010621 and rs2297441) may be associated with the risk of stroke in the Chinese Han population.

Stroke is a multifactorial disease associated with a variety of factors. Among them, the growth of age and stroke risk is positively correlated. Vascular cell senescence contributes to endothelial dysfunction [19], which can cause atherosclerotic plaque progression [20], further resulting in stroke [21]. It has been demonstrated in previous studies that telomere length shortening is associated with stroke [7–9]. The telomere length decreases continuously with aging due to the decrease of telomerase activity [22].The length of telomeres is also affected by many factors, including Eating habits and lifestyles [23], body mass index (BMI) and smoking [24], which genetic factors in different races occupied 36% −84% [25]. *RTEL1* is the essential helicase for telomere maintenance and the regulation of homologous recombination, and plays an important role in maintaining telomere length [26]. The *RTEL1* gene, as an important gene for maintaining telomere length, has been confirmed to be associated with telomere-related diseases such as Hoyeraal Hreidarsson Syndrome, glioma, lung cancer and atopic dermatitis [15–18], But so far, no studies have investigated the association between SNPs in the RTEL1 gene and the risk of stroke. Based on our experimental results, we conclude that perhaps the SNPs in the *RTEL1* gene can cause cell apoptosis by influencing the length of telomere, and then causes endothelial dysfunction, and finally affects stroke.

Recent studies have reported that the genetic polymorphisms of telomere-related genes such as ACYP2, TSPYL6, and TERT are associated with stroke by affecting telomere length [10, 11], including rs11125529, rs12615793, rs843711, rs11896604, rs843706 within both ACYP2 and TSPYL6, rs17045754 in ACYP2 gene, and rs2736122, rs2853668 in TERT gene. In our experimental results, We found that four SNPs (Rs2297441, Rs6089953, Rs6010620, and Rs6010621) were associated with the risk of stroke in this population. It may be possible to show that the mechanism of the SNPs in the *RTEL1* gene impact on stroke may be the same as other telomere - related genes. Because in previous studies there was a lack of research and reporting on the association between the SNPs in the *RTEL1* gene and Stroke, we have no way to compare with other data. The exact mechanism for explaining the relationship between the SNPs in the *RTEL1* gene and stroke cannot be determined from our study alone, we should do further functional experiments to verify.

There are several limitations of this study. First, the sample size is not large enough, Second, the clinical subtypes of stroke were not considered in this study. In addition, some influencing factors such as eating habits, living environment, and Smoking history are not considered. These limitations will be addressed in future research.

## MATERIALS AND METHODS

### Study participants

A total of 400 patients with stroke and 395 healthy individuals were enrolled in the study. The characteristics of the participants are shown in Table 1. All participants are genetically unrelated Han Chinese. The stroke status of each case was determined by Magnetic Resonance Imaging and their clinical records as confirmed by two clinicians. Healthy controls without history of stroke and neurological impairments were recruited from healthcare center of our hospital. This study was conducted in accordance with the Chinese Department of Health and Human Services regulations for the protection of human research subjects. Informed consent was obtained from all participants.

### SNP selection and genotyping

We selected five SNPs from previously reported RTEL1 gene polymorphisms, and matched SNPs with MAF > 5% in the HapMap Asian population selected for association analysis [16, 27, 28]. Venous blood samples (5 mL) were collected from each Participant during a laboratory examination. DNA was extracted from whole blood samples using the Gold Mag-Mini Whole Blood Genomic DNA Purification Kit (version 3.0; TaKaRa, Japan) [29]. The DNA concentration was measured by spectrometry (DU530 UV/VIS spectrophotometer, Beckman Instruments, Fullerton, CA, USA). The Sequenom MassARRAY Assay Design 3.0 software (Sequenom, Inc, San Diego, CA, USA) was used to design the multiplexed SNP Mass EXTEND assay. Genotyping was performed using a Sequenom MassARRAY RS1000 (Sequenom, Inc.) according to the manufacturerfacturerufa [30]. The SequenomTyper 4.0 Software 4.Sequenom, Inc.) was used to manage and analyze the data [31]. Based on these results, the following five SNPs were selected: rs6089953, rs6010620, rs6010621, rs4809324, and rs2297441. The SNP data are shown in Table 3.

### Statistical analysis

Pearson’s χ^2^ test and Welch’s *t* test were used to evaluate the differences in the demographic characteristics between the cases and controls [32]. The Hardy-Weinberg equilibrium of each SNP was assessed in order to compare the expected frequencies of the genotypes in the control patients. All of the minor alleles were regarded as risk alleles for stroke susceptibility. To evaluate associations between the SNPs and risk of stroke in the four models (genotype, dominant, recessive, and additive), ORs and 95% CIs were calculated using unconditional logistic regression analysis [33]. In multivariate analyses, unconditional logistic regression was used to assess the association between each SNP and the risk of stroke [33]. Linkage disequilibrium analysis and SNP haplotypes were analyzed using the Haploview software package (version 4.2) and the SHEsi software platform (http://www.nhgg.org/analysis/) [34]. All statistical analyses were performed using the SPSS version 17.0 statistical package (SPSS, Chicago, IL, USA) and Microsoft Excel. A *p* < 0.05 was considered statistically significant and all statistical tests were two-sided.

## CONCLUSIONS

In summary, we have identified four new associations between the SNPs (Rs6089953, Rs6010620, Rs6010621 and Rs2297441) in *RTEL1* gene and stroke. Our results suggest that these SNPs may serve as molecular markers of stroke susceptibility for primary prevention of stroke during clinical treatment. At the same time, this study provides a new idea for future research on association between single nucleotide polymorphisms and Stroke.
